# Allocation of the ICU wards according to the patient’s infection condition: a measure to improve antibiotic resistance

**DOI:** 10.1186/s13054-020-03192-y

**Published:** 2020-07-29

**Authors:** Yefei Zhan, Peifu Chen, Jieqiong Chen, Hua Wang, Zhaojun Xu, Yu Chen

**Affiliations:** 1 Department of Intensive Care Unit, Hwa Mei Hospital, University of Chinese Academy of Sciences, No.41, Northwest Street, Ningbo, 315010 Zhejiang China; 2 Ningbo Institute of Life and Health Industry, University of Chinese Academy of Sciences, Ningbo, China

**Keywords:** Intensive care unit, Nosocomial infections, Multidrug-resistant organism, Environmental contamination, Isolation precaution

Pathogens are known to survive on surfaces in health care environments despite routine cleaning and nearly always cause nosocomial infections [1–3]. The number of health care-associated infections is progressively increasing and the extent of multidrug-resistant organisms (MDROs) in medical institutions is of worldwide concern and continues to challenge infection control [4, 5]. The aim of our study was to find the simplest and most effective way to reduce the spread of MDROs by using “infection grade ward isolation” (IGWI).

We conducted a 1-year prospective study to evaluate the impact of the IGWI method on MDRO environmental contamination and colonization in the intensive care unit (ICU). The study comprised a 3-month baseline period and a 9-month intervention period. The baseline period ranged from March 1, 2018, to May 31, 2018. There was no difference between ICU-1 (10 single rooms and 5 double rooms with 20 beds) and ICU-2 (newly built, 2 multi-bed rooms with 12 beds) in terms of patient admission, medication, and ward disinfection. The intervention period lasted from June 1, 2018, to February 28, 2019. During this period, IGWI was implemented; patients with MDRO infections or who used more than a third-generation cephalosporins or other strong antibiotics were not allowed into ICU-2, and patients in ICU-2 needing advanced antibiotics or with newly found MDROs were quickly transferred to ICU-1. Both wards had noninterchangeable equipment, work clothes, and noninteracting staff.

Samples from five different surfaces—an air conditioning vent, an oxygenation probe, an intravenous pump, a bed rail button, and a sheet (from around the perineal area of the patient)—from 32 beds (20 beds in ICU-1 and 12 beds in ICU-2) were collected on sterile rayon swabs each month, which were then cultured for the presence of MDROs.

We collected 1920 swabs over 1 year—1200 from ICU-1 and 720 from ICU-2—480 were collected during the baseline period and 1440 during the intervention period. Of these 1920 swabs, 73 were MDRO positive (Table 1). During the baseline period, the MDRO detection rate was 6.7 ~ 10.0% (Table 2), and there was no difference in the incidence of MDROs (6.7 vs. 10.0%, *P* = 0.109, Table 2) between ICU-2 and ICU-1. During the intervention period using the IGWI method, the MDRO overall detection rate in for both ICU wards was reduced to 0 ~ 3.4% (Table 2). The positive rate of MDROs in the sample cultures was greater in ICU-1 than in ICU-2 (3.4 vs. 0%, *P* = 0.007). Both wards had a greater proportion of positive results for MDROs during the baseline stage than after the intervention (ICU-1 10 vs. 3.4%, respectively, *P* < 0.001; ICU-2 6.7 vs. 0%, respectively, *P* < 0.001; Table 2).

**Table 1:** MDROs found from the five contact surface samples

ICU wards	Period	Time	MDROs	CRAB	CRE	ESBLs	MDR-AB	MRSA	MRSE
			(***n*** = 73)	(Bed No.+ Surface)	(Bed No.+ Surface)	(Bed No.+ Surface)	(Bed No.+ Surface)	(Bed No.+ Surface)	(Bed No.+ Surface)
**ICU-1**	**Baseline**	2018, Mar	**9**		17I	2B	2A, 7S, 20B, 20A, 20I	13B	12S
		2018, Apr	**11**			12S	7I, 17I, 16S, 5A, 17S, 4I, 13O		1A, 10S, 13A,
		2018, May	**10**				17A, 17S, 12S, 12B, 17B, 7I, 7S, 7A, 17O, 11B		
	**Intervention**	2018, Jun	**4**		19B		15B, 18S, 17B,		
		2018, Jul	**7**		14S		7S, 6S, 1O, 18A	2O, 1A,	
		2018, Aug	**1**					2A	
		2018, Sep	**2**				16A, 14A		
		2018, Oct	**4**			5A		2I, 18O, 2S	
		2018, Nov	**2**				16A	2S	
		2018, Dec	**1**		3S				
		2019, Jan	**3**	12A		9I		20B	
		2019, Feb	**7**		5S	10S	9B, 1A		4S, 11A, 3A
**ICU-2**	**Baseline**	2018, Mar	**7**			6O			4O, 6B, 6S, 8S, 11B, 11S
		2018, Apr	**4**			4B			4S, 6B, 9B
		2018, May	**1**				8I		
	**Intervention**	2018, Jun	**0**						
		2018, Jul	**0**						
		2018, Aug	**0**						
		2018, Sep	**0**						
		2018, Oct	**0**						
		2018, Nov	**0**						
		2018, Dec	**0**						
		2019, Jan	**0**						
		2019, Feb	**0**						

*MDROs* multidrug-resistant organisms, *ICU* intensive care unit, *MDR-AB* multidrug-resistant *Acinetobacter baumannii*, *CRE* carbapenem-resistant enterobacteria, *CRAB* carbapenem-resistant *A. baumannii*, *MRSA* methicillin-resistant *Staphylococcus aureus*, *MRSE* methicillin-resistant *S. epidermidis*, *ESBLs* extended-spectrum β-lactamase (ESBL)–producing *Escherichia coli* (*E. coli*) and *Klebsiella pneumoniae* (*K. pneumoniae*), *A* air conditioning vent, *B* bed rail button, *O* oxygenation probe, *I* intravenous pump, *S* sheet (perineal area)

**Table 2:** Positive cultures of MDROs from samples taken from contact surfaces during the 1-year study

Time	MDROs [n (%)]		***P*** value
	ICU-1 [n/N (n%), *N* = 100]	ICU-2 [n/N (n%), *N* = 60]	
**Baseline**	**30/300 (10.0%)**	**12/180 (6.7%)**	0.109
2018, Mar	9/100 (9.0%)	7/60 (11.7%)	
2018, Apr	11/100 (11.0%)	4/60 (6.7%)	
2018, May	10/100 (10.0%)	1/60 (1.7%)	
**Intervention**	**31/900 (3.4%)**	**0/540 (0.0%)**	0.007
2018, Jun	4/100 (4.0%)	0/60 (0.0%)	
2018, Jul	7/100 (7.0%)	0/60 (0.0%)	
2018, Aug	1/100 (1.0%)	0/60 (0.0%)	
2018, Sep	2/100 (2.0%)	0/60 (0.0%)	
2018, Oct	4/100 (4.0%)	0/60 (0.0%)	
2018, Nov	2/100 (2.0%)	0/60 (0.0%)	
2018, Dec	1/100 (1.0%)	0/60 (0.0%)	
2019, Jan	3/100 (3.0%)	0/60 (0.0%)	
2019, Feb	7/100 (7.0%)	0/60 (0.0%)	
***P*value**	<0.001	<0.001	

*ICU* intensive care unit, *MDROs* multidrug-resistant organisms

We found a very effective technique—the IGWI method—that in essence helps prevent non-MDRO-infected patients who enter the ICU from coming into contact with MDRO environment. The IGWI method has two criteria for distinguishing ICU patients—whether the patients are infected by MDROs or are using or in need of advanced antibiotics.

Currently, ICU departments are always divided into different wards to receive patients with different diseases. Many patients who are not infected with drug-resistant bacteria could be exposed to a bad ICU environment and become innocent victims of environmental contamination. For quarantined patients, the unconscious negligence of a person or a link often results in quarantine failure [6] and risks drug-resistant bacterial infection of those patients with nonresistant bacterial infections [3]. This study found that breaking the traditional protocol and using the IGWI method to differentiate patients in different ICU wards would greatly reduce the environmental MDRO infection rate.

## Data Availability

All collected data were listed in an anonymous database. The dataset is not available but can be requested from the corresponding author.

## Abbreviations

MDROs: Multidrug-resistant organisms
IGWI: Infection grade ward isolation
ICU: Intensive care unit
